# Role of the Endocannabinoid/Endovanilloid System in the Modulation of Osteoclast Activity in Paget’s Disease of Bone

**DOI:** 10.3390/ijms221810158

**Published:** 2021-09-21

**Authors:** Marco Paoletta, Antimo Moretti, Sara Liguori, Alessandra Di Paola, Chiara Tortora, Maura Argenziano, Francesca Rossi, Giovanni Iolascon

**Affiliations:** 1 Department of Medical and Surgical Specialties and Dentistry, University of Campania “Luigi Vanvitelli”, 80138 Naples, Italy; marco.paoletta@unicampania.it (M.P.); sara.liguori@unicampania.it (S.L.); giovanni.iolascon@unicampania.it (G.I.); 2 Department of Experimental Medicine, University of Campania “Luigi Vanvitelli”, S. Maria di Costantinopoli 16, 80138 Naples, Italy; alessandra.dipaola92@gmail.com; 3 Department of Woman, Child and General and Specialist Surgery, University of Campania “Luigi Vanvitelli”, L. De Crecchio 4, 80138 Naples, Italy; chiara.tortora@unicampania.it (C.T.); maurargenziano@gmail.com (M.A.); francesca.rossi@unicampania.it (F.R.)

**Keywords:** cannabinoid receptor type 2, Paget’s disease of the bone, endocannabinoid/endovanilloid system, pain, bone diseases, metabolic

## Abstract

The role of the endocannabinoid/endovanilloid (EC/EV) system in bone metabolism has recently received attention. Current literature evidences the modulation of osteoclasts and osteoblasts through the activation or inhibition of cannabinoid receptors in various pathological conditions with secondary involvement of bone tissue. However, this role is still unclear in primary bone diseases. Paget’s disease of the bone (PDB) could be considered a disease model for analyzing the role of the EC/EV system on osteoclasts (OCs), speculating the potential use of specific agents targeting this system for managing metabolic bone disorders. The aim of the study is to analyze OCs expression of EC/EV system in patients with PDB and to compare OCs activity between this population and healthy people. Finally, we investigate whether specific agents targeting EC/EV systems are able to modulate OCs activity in this metabolic bone disorder. We found a significant increase in cannabinoid receptor type 2 (CB2) protein expression in patients with PDB, compared to healthy controls. Moreover, we found a significant reduction in multi-nucleated tartrate-resistant acid phosphatase (TRAP)–positive OCs and resorption areas after treatment with JWH-133. CB2 could be a molecular target for reducing the activity of OCs in PDB, opening new therapeutic scenarios for the management of this condition.

## 1. Introduction

Paget’s disease of bone (PDB) is a skeletal disorder that was described for the first time by Sir James Paget in 1887. It is a chronic metabolic disease characterized by a focal osteolytic lesion involving one (monostotic form) or more skeletal sites (polyostotic form). Paget’s disease of bone is most common in older individuals, and may lead to bone pain, deformities, and fractures. The etiology is still unclear but a viral infection, in particular by paramyxoviruses, is supposed, considering the viral-like inclusions in the nuclei of osteoclasts (OCs) in affected patients [1]. This hypothesis is also consistent with some epidemiological observations, taking into account a progressive decline of PDB prevalence in accordance with infection limitations during the last decades [2]. Even though the exact pathophysiological mechanisms of PDB are still debated, it has been proved an aberrant bone remodeling method mainly due to OCs dysfunction. Indeed, skeletal lesions are caused by increased OCs activity associated to subsequent disorganized bone formation [3]. Considering the available therapeutic options, bisphosphonates (BPs) are currently recommended for the treatment of patients with active PDB [4]. In particular, intravenous zoledronic acid has a greater effect on bone turnover, with prolonged benefit up to 3 years [5]. At the same time, intravenous or intramuscular neridronic acid are associated with significant clinical remission, including pain relief, and biochemical response [6,7]. Emerging evidence suggests that agents modulating the endocannabinoid/endovanilloid (EC/EV) system affects bone remodeling in animal models and humans. Therefore, cannabinoids could have an ancillary role for the treatment of PDB.

The endocannabinoid system is mainly composed of endogenous cannabinoid ligands and two receptors: cannabinoid receptor type 1 (CB1) and type 2 (CB2) [8]. Endocannabinoids could interact with other types of receptors, including the transient receptor potential cation channel subfamily V member 1 (TRPV1) [9]. In particular, CB1 and TRPV1 receptors’ stimulation seems to have an activating role on OCs, while CB2 stimulation results in their inhibition, balancing bone mineralization and remodeling [10]. CB2, a G protein-coupled receptor, is a member of the endocannabinoid system predominantly expressed in peripheral tissues, such as bone. Through the activation of the ERK-AP1 pathway via CB2 receptor stimulation, it regulates osteoclasts’ differentiation, survival and function [11,12]. Although preclinical studies are promising, the use of CB2 agonists in clinical practice has not yet been investigated.

Based on these premises, we aim to analyze OCs expression of EC/EV system in patients with PDB and to compare OCs activity between this population and healthy people. Finally, we investigate whether specific agents targeting the EC/EV system are able to modulate OCs activity in this metabolic bone disorder.

## 2. Results

### 2.1. Patients

Four women with PDB and four age-matched controls were enrolled. Of the four women with PDB, three had polyostotic form, while one was affected by the monostotic form.

### 2.2. Cell Characterization

After 21 days of differentiation, cells obtained from both healthy donors and PDB patients were analyzed. Most of cells were multinucleated and TRAP-positive and therefore, identified as mature OCs.

**Figure 1:**
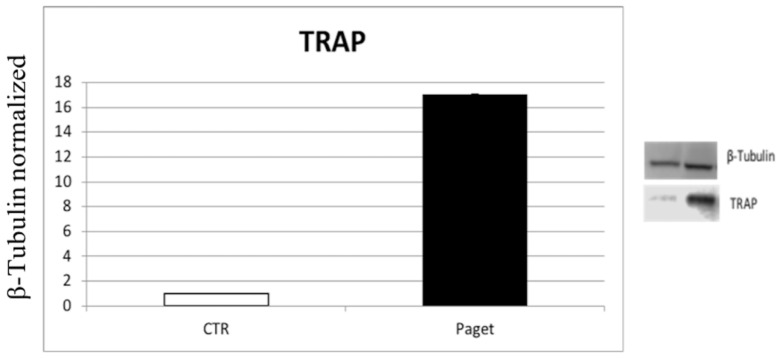
Comparison of the expression of the TRAP enzyme between OCs of 2 subjects with PDB and 2 healthy ones. The most representative images are displayed. The relative quantification for TRAP expression normalized for the housekeeping protein β-Tubulin is presented in histograms as the mean ± SD of independent experiments of each individual sample.

Using Western blot, a significantly higher expression of TRAP enzyme levels was observed in patients with PDB, compared to healthy donors. These data confirm high osteoclasts activity usually found in patients with PDB associated to high bone resorption activity (Figure 1).

### 2.3. Characterization of the Endocannabinoid/Endovanilloid System

**Figure 2:**
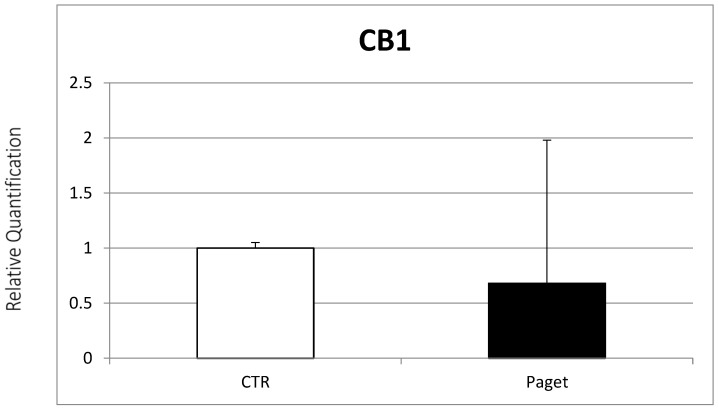
Comparison between CB1 mRNA expression evaluated with RT-PCR from 2 OCs of subjects with PDB and 2 healthy controls. Results were normalized for the housekeeping gene β-Actin and were showed as mean ± SD of three independent experiments on each individual sample.

**Figure 3:**
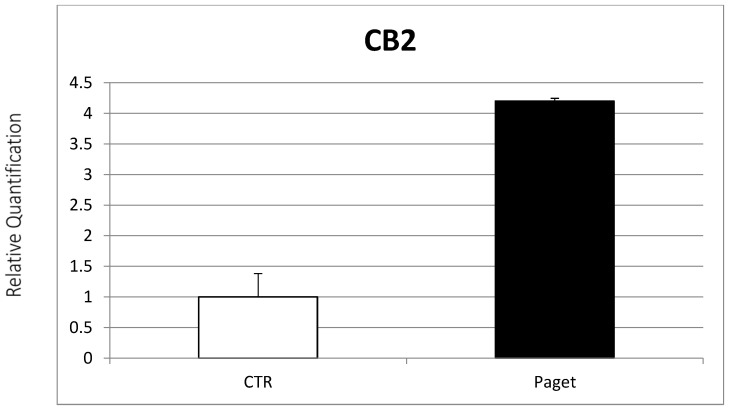
Comparison between CB2 mRNA expression evaluated with RT-PCR from 2 OCs of subjects with PDB and 2 healthy controls. Results were normalized for the housekeeping gene β-Actin and were showed as mean ± SD of three independent experiments on each individual sample.

**Figure 4:**
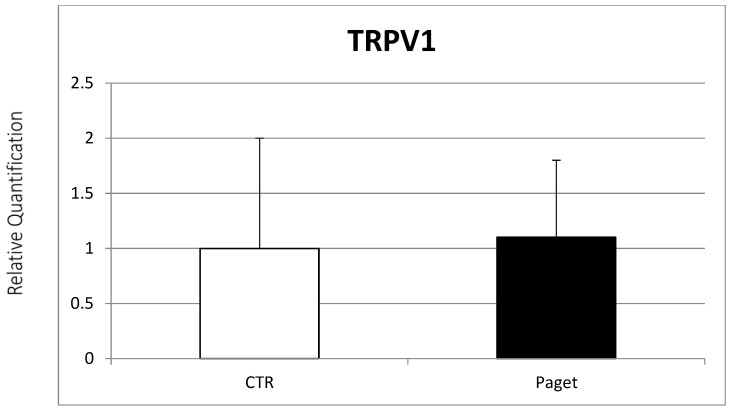
Comparison between TRPV1 mRNA expression evaluated with RT-PCR from 2 OCs of subjects with PDB and 2 healthy controls. Results were normalized for the housekeeping gene β-Actin and were showed as mean ± SD of three independent experiments on each individual sample.

Osteoclasts’ expression of the EC/EV system found in patients with PDB was subsequently evaluated. Real-time PCR evidenced the expression of TRPV1, CB1 and CB2 receptors from OCs of PDB patients. In particular, lower CB1 gene expression (Figure 2) and a significant increase in CB2 gene expression were found in OCs of patients with PDB, compared to healthy controls (Figure 3). On the other side, we observed no between-group difference for TRPV1 gene expression (Figure 4).

**Figure 5:**
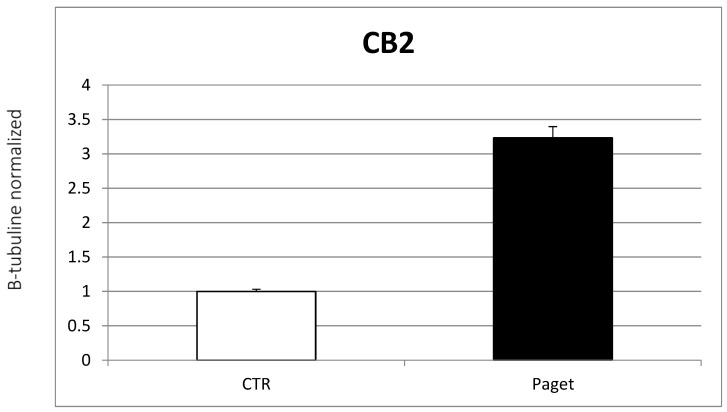
Comparison between CB2 protein expression at Western blotting from 2 OCs of subjects with PDB and 2 healthy controls. The most representative images are displayed. The relative quantification for TRAP expression normalized for the housekeeping protein β-Tubulin is presented in histograms as the mean ± SD of independent experiments of each individual sample.

Western blot analysis evidenced a significant increase in CB2 protein expression in patients with PDB, compared with healthy controls (Figure 5).

### 2.4. Effects of Stimulation of the Endocannabinoid/Endovanilloid System in Osteoclasts Obtained from Patients with PDB

#### 2.4.1. TRAP Assay

We evaluated OCs number and activation, using TRAP Assay after treatment with JWH-133 (selective CB2 agonist), AM630 (reverse CB2 agonist), RTX (selective TRPV1 agonist) and I-RTX (TRPV1 antagonist). We found a significant reduction in multi-nucleated TRAP-positive OCs (*n* ≥ 3) after treatment with JWH-133, while the treatment with AM630 showed no significant changes.

**Figure 6:**
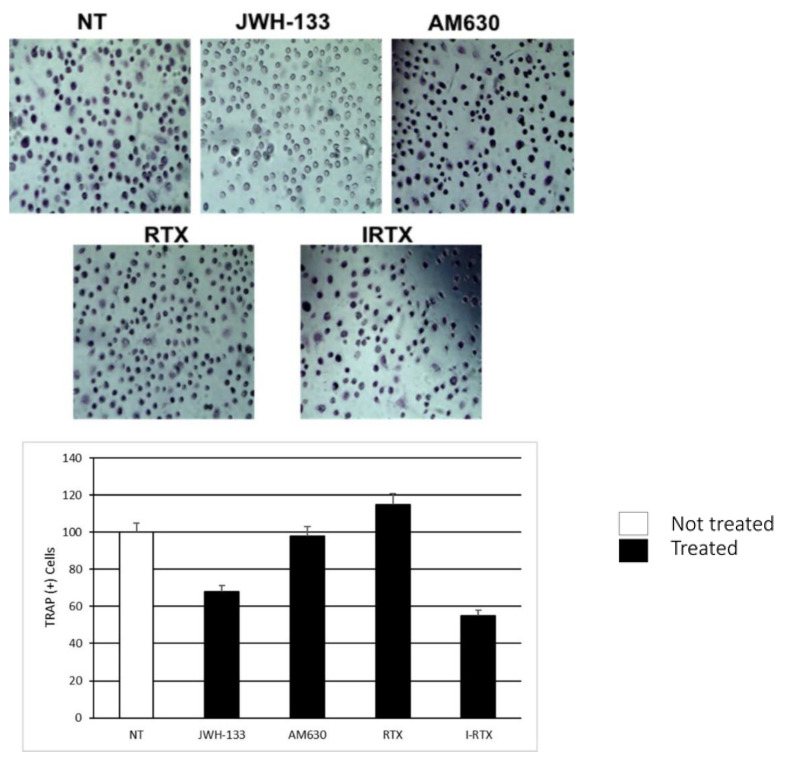
Comparison of the TRAP assay, performed on 2 OCs of patients with PBD, in the untreated (NT), JWH-133 AM630, RTX and IRTX treated samples. The most representative images are displayed. The percentage number of TRAP (+) cells with respect to the total cells number for each sample is presented in the histogram as mean ± SD.

A significant increase in the number of active OCs was found after treatment with RTX. Conversely, treatment with I-RTX induced a significant reduction in the number of OCs in patients with PDB, compared to healthy controls (Figure 6).

#### 2.4.2. Bone Resorption Assay

**Figure 7:**
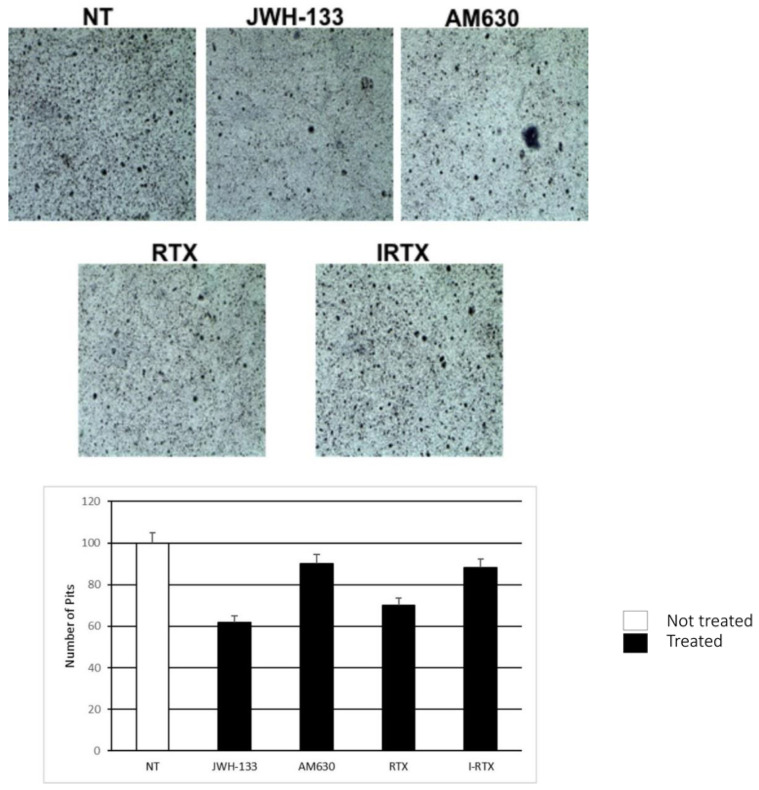
Comparison of bone resorption assay, performed on 2 OCs of patients with PBD, in the untreated (NT), JWH-133 AM630, RTX and IRTX treated samples. The most representative images are displayed. The percentage number of reabsorption pits is presented in histograms as mean ± SD.

Bone resorption assay showed a significant reduction in the resorption areas in in vitro samples treated with JWH-133, compared to untreated controls. Otherwise, there was a significant increase in the number and size of bone resorption areas after treatment with AM630. However, treatment with I-RTX did not show a significant reduction in the bone resorption area, compared to untreated controls (Figure 7).

## 3. Discussion

Paget’s disease of bone (PDB) is characterized by aberrant bone remodeling with disproportionate increase in osteoclasts (OCs) activity, compared to OB activity [13], which leads to overall bone resorption, causing typical skeletal lesions. The mechanisms underlying the increased number and activity of OCs in the PDB are still largely unknown. Our results show an important overexpression of the TRAP enzyme in OCs of PDB patients in comparison with healthy controls, confirming the high erosive activity of OCs that compromises bone quality [14]. It was demonstrated that osteoclasts express EC/EV receptors in both healthy subjects and PDB patients and that they are involved in the regulation of bone metabolism [12,13,14,15].

Both TRPV1 and CB1 significantly stimulate OCs-mediated bone resorption. In particular, genetic deletion or pharmacological inhibition of TRPV1 significantly reduce OCs’ activity [16]. Similarly, also CB1 knockout mice were completely resistant to ovariectomy-induced trabecular bone loss, compared to the wild type [12]. Even though CB1 receptor is ubiquitously expressed, it is principally localized in the brain and peripheral nerves; therefore, targeting it could cause psychotropic side effects [17]. Overexpression of CB2 receptor by OCs inhibits bone resorption [11]. Rossi et al. demonstrated that cannabinoid and vanilloid receptors are co-expressed in human OCs, suggesting their modulating activity of mineralization and bone resorption [15,16].

Considering these observations, the administration of compounds activating CB2 receptors and/or antagonizing/desensitizing TRPV1 channels might have potential benefits for managing metabolic bone disorders in which an imbalance of coupling (i.e., OB/OC activity) is observed.

### 3.1. Osteoclast Expression of EC/EV System in Patients with PDB

In our study, we demonstrated the expression of EC/EV system receptors in OCs of PDB patients. In particular, these patients show lower CB1 expression, but similar TRPV1 receptor levels, compared to healthy controls. On the other hand, increased CB2 gene expression was found in PDB patients. Our results seem to be in contrast with data supporting the “anti-osteoclastogenic” activity of CB2 receptor. In fact, it can be possible that other genomic pathways and environmental factors may influence the bone metabolism as well as CB2 receptor expression in PDB.

A possible mechanism could be the greater sensitivity of the OC precursors of subjects with PDB to biochemical factors, such as 1,25(OH)vitamin D3, which would cause an increased number of OCs [18]. Moreover, additional stimulating factors seem to have a significant influence on OCs activity in PDB. It was proposed an osteoimmunological pathway as a potential underlying mechanism for PDB [19]. Increased levels of M-CSF and IL-6 stimulating OCs formation and activation were found in untreated PDB patients. The role of these immunological factors was confirmed by the inhibition of OCs differentiation and proliferation by administering dexamethasone and monoclonal antibodies to M-CSF and IL-6 [20]. In this context, considering the emerging effects on the immune system of EC, it is possible to speculate a modulating role of bone remodeling through this pathway. Both innate and adaptive immune cells express CB2 receptors, and their activation induces apoptosis and inhibits autoantibodies synthesis and pro-inflammatory cytokine expression. These effects support the role of CB2 as a potential target for reducing inflammation and regulating the immune response [21]. In our population, we found increased CB2 receptor expression, contrary to what was expected. It could be speculated a compensatory mechanism of OCs to counteract inflammation. Therefore, using agents stimulating CB2 receptors might have a dual effect by inhibiting both OCs activity and immune response.

In our opinion, cannabinoids might not have the same role in the PDB as in healthy bone or in other metabolic bone disorders. For the latter two, OCs are not involved in the main pathophysiological mechanisms of disease, but they respond to stimulating endocrine and/or paracrine factors. In PDB, impaired function of OCs is the key mechanism in the development of the disease, and we could speculate that also the cannabinoid pathway is modified in these cells. Recently, it was demonstrated that two micro-RNAs (miR-146a-3p and miR-155-5p) are significantly reduced in pagetic OCs. These miRs have a silencing effect on the expression of genes, such as mTOR and Akt, involved in the formation, activation and functions of OCs [22].

It was demonstrated the high levels of CB2 expression in patients with advanced non-small-cell lung cancer and, conversely, the low expression of CB2 suppresses the Akt/mTOR pathway [23]. These findings let us to speculate that, at least in some stages of the PDB, an increased overexpression of CB2 receptor could even have a paradoxical effect, being associated with a stimulation of OCs. However, further studies are needed to confirm this hypothesis.

### 3.2. Modulation of OCs Activity through Agents Addressing EC/EV System

In our study, we observed a dramatic reduction in the TRAP+ OCs number after treatment with JWH-133, a selective agonist of CB2 receptor. This result supports the role of this receptor as an inhibitor of OCs maturation and activation. On the contrary, the administration of AM630, an inverse agonist of CB2 receptor, resulted in increased stimulation of OCs as highlighted by TRAP activation. Our findings are consistent with those already reported in previous studies on the effects of CB2 stimulation in postmenopausal women [16] and in patients with osteosarcoma [24]. This evidence suggests that targeting the CB2 receptor might regulate bone turnover, even in patients with PDB.

Similarly, the TRAP assay suggests that the administration of IRTX, a competitive antagonist of the TRPV1 receptor, significantly reduces the number of OCs, but not their activity. These data confirm the possible role of the vanilloid receptor in modulating OCs activity.

The bone resorption assay confirmed the modulation of OCs activity through the stimulation or inhibition of EC system in vitro. Our study showed significant reduction in the number and size of the bone resorption areas in samples treated with JWH-133, compared to the untreated controls. The number and width of the resorption areas are directly proportional to the OCs activation, so our finding suggests that the activation of CB2 has a crucial effect in reducing OCs activity also in PDB. On the contrary, the bone resorption assay did not show any difference between the sample treated with IRTX, and controls. This result would confirm the limited role of the vanilloid receptor in the modulation of OCs activity in subjects with PDB.

In patients with PDB, the exacerbation of pain is one of the most referred symptoms that has a significant impact on the functioning and quality of life [25]. Although bone pain is commonly reported in PDB patients because of impaired bone remodeling, disorganization of the bone architecture and microfracture, due to mechanical stress, pain could also arise from the involvement of nerves in bone with consequent development of peripheral and central neuropathic pain. Pain modulation is a further effect of the activation of CB2 receptor. Although several CB2 agonists have been investigated as analgesics, to date, there is no unanimous consensus to support their use in clinical practice. It was demonstrated that intrathecal injection of agents activating CB2 inhibits glial cells and downregulates the expression of IL-1β and IL-6, reducing bone cancer pain [26]. A possible explanation of this effect seems to be an improvement of the impaired autophagy flux by glia-derived inflammatory mediators by administering CB2 agonists. The global effect of stimulating the CB2 receptor is a modulation of neuropathic pain, particularly in terms of central sensitization and pain behavior [27].

Our study has some limitations. First, we have a small sample size that does not allow to draw definitive results on the role of the EC/EV system in subjects affected by PDB. Moreover, we have not followed the patients over time to evaluate potential changes in the response of the CB2 modulator in the various stages of the disease. Nevertheless, it is possible to have a small number of patients when investigating a new treatment approach, in particular in a non-common condition, such as PDB. Certainly, our results should be validated in a larger cohort. Finally, we do not have blood chemistry data and the presence of bone loss at the diagnosis.

## 4. Materials and Methods

Patients: The study was conducted at the Department of Medical and Surgical Specialties and Dentistry, and at the Department of Women, Children and General and Specialized Surgery of the University of Campania “Luigi Vanvitelli”.

We enrolled four women (mean age 61 ± 14.07 years) affected by PDB, presenting the following characteristics: (1) exacerbation of clinical symptoms attributable to PDB (i.e., bone pain); (2) typical radiological findings of PDB, such as osteolytic areas, cortical thickening, loss of distinction between cortex and medulla, trabecular thickening, osteosclerosis, bone expansion and bone deformity; (3) biochemical findings, in particular, increased serum total alkaline phosphatase (ALP) and bone alkaline phosphatase (bALP) at least three fold over the normal ranges. We excluded subjects treated with BPs or steroids/estrogens in the 3 year period prior to recruitment. All patients provided informed written consent to participate in the study. Enrolled patients were subjected to blood sampling, and the following in vitro assays were performed. As the control group, we recruited three healthy, age-matched volunteer women (mean aged 58 ± 6.24 years). All procedures performed in this study were in accordance with the Helsinki Declaration of Principles, the Italian National Legislation, and the Ethics Committee of the University of Campania “Luigi Vanvitelli” (committee’s reference number: 266, approved on 18 September 2020). Informed consent was obtained from all subjects involved in the study.

Human cell cultures: Osteoclasts were differentiated from the peripheral blood mononuclear cells (PBMC) of enrolled healthy donors and PDB patients. PBMCs were isolated by centrifugation over Histopaque 1077 density gradient (Sigma Chemical, St Louis, MO, USA), diluted at 1 × 106 cells/mL in α-Minimal Essential Medium (α-MEM)) supplemented with 10% fetal bovine serum (FBS) (Euroclone, Siziano, Italy), 100 IU/mL of penicillin, 100 g/mL of streptomycin (Gibco Limited, Uxbridge, UK), l-glutamine (Sigma Aldrich, Milan, Italy), plated in 24-well plates and incubated at 37 °C in a humidified atmosphere containing 5% CO_2_ for 21 days in the presence of 25 ng/mL of recombinant human macrophage-colony stimulating factor (rhM-CSF) (Peprotech, London, UK) and 50 ng/mL of receptor activator of nuclear factor kappa B ligand (RANK-L) (Peprotech, London, UK). The culture medium was replaced every three days with fresh medium supplemented with the agents described above. After 21 days, mature OCs were identified as tartrate-resistant acid phosphatase (TRAP)–positive multinucleated cells. Mature OCs were collected for the extraction of mRNA and proteins.

mRNA Extraction and Real Time-PCR:

The total RNA from MSC cultures was extracted, using Qiazol^®^ (Qiagen, Hilden, Germany) following the manufacturer’s instructions. EasyScript™ cDNA Synthesis Kit (abm, Foster City, CA, USA.) was used to synthesize from approximately 1000 ng of mRNA, the first-strand cDNA. The transcript levels of CB2 were detected by RT-qPCR, using a CFX96 Real-Time PCR system (Bio-Rad, Hercules, CA, USA.), using I-Taq Universal SYBR^®^ Green Master Mix (Bio-Rad). The cycling conditions were 10 min at 95 °C (initial denaturation), followed by 40 cycles of 15 s at 94 °C (denaturation) and 1 min at 68 °C (annealing/extension/data collection). The β-actin gene served as the reference gene for the normalization of the real-time PCR products. The PCR primers used to detect each gene were designed, using the Primer 3 program and synthesized by Sigma Aldrich (CB1_F 5′-CGTCTGAGGATGGGAAGGTA-3′, CB1_R 5′-ACCAGGGTCTTGGCTAACCT-3′; CB2_F 5′-AAGGCTGTCTTCCTGCTGAA-3′, CB2_R 5′-CACAGAGGCTGTGAAGGTCA-3; TRPV1_F 5′-CTGCAGAAGAGCAAGAAGCA-3′, TRPV1_R 5′-ATGGC TTTCAGCAGACAGGT-3′ β-Actin_F 5′-GCGAGAAGATGACCCAGATC-3′, β-Actin_R 5′-GGA TAGCACAGCCTGGATAG-3′). We performed the assays in technical triplicate and tested the linearity and efficiency of the experiments over dilutions of cDNA, including five orders of greatness. To confirm the specificity of the reactions, we performed the dissociation curve analysis of amplification products. To analyze the data and achieve the relative gene expression levels, we used the 2^−∆∆Ct^ method.

Evaluation of OCs activity: TRAP assay and bone resorption assay. The TRAP assay was performed, using the TRACP and ALP Assay Kit (Takara Bio, Shiga, Japan) in triplicate on each individual sample. After cell fixation, performed with citrate buffer at a pH of 5.4, containing 60% acetone and 10% methanol for 5 min, at room temperature, 50 μL of chromogenic substrate solution (naphtol-AS-BI-phosphate substrate) was added (fast red violet LB) containing 0.1 vol of sodium tartrate. The TRAP enzyme cleaved the substrate, forming a compound that gives a purplish red color that can be detected with an optical microscope (Nikon Eclipse TS100, Nikon Instruments, Badhoevedorp, the Netherlands). Each experiment has a positive and a negative control to ensure the functionality of the test. The bone resorption assay was performed, using a commercially available bone resorption assay kit (CosMo Bio, Tokyo, Japan) in triplicate on each individual sample. Osteoclasts were placed in 24-well plates coated with calcium phosphate. The wells were washed on day 21 with a 5% NaOCl solution to remove the cells, and the resorption area was viewed under an optical microscope (Nikon Eclipse TS100, Nikon Instruments, Badhoevedorp, the Netherlands) and measured with dedicated software.

The bone resorption assay was performed, using commercially available kits (CosMo Bio, Tokyo, Japan). Osteoclasts were differentiated from PBMCs from PDB patients and healthy donors into 24 cells, containing calcium phosphate. The RANK-L was used at the dosage of 100 ng/mL. JWH-133 (100 mM), AM630 (10 μM), RTX and IRTX were added on day 12 for 48 h. On day 14, the cells were removed using 5% sodium hypochlorite to visualize and count the resorption pits with the optical microscope (Nikon Eclipse TS100, Nikon Instruments, Amsterdam, the Netherlands).

Western blot: Proteins were extracted from OCs, using a lysis buffer in order to verify the expression of TRAP and CB2 proteins by Western blot from independent experiments on each individual sample. The proteins were separated by running on a polyacrylamide gel and transferred to the membrane. Membranes were incubated for 4 h at 4 °C with rabbit polyclonal anti-TRAP (SC-28204, dilution 1:200; Santa Cruz, CA, USA), a rabbit polyclonal anti-CB2 antibody (1:500 dilution; Abcam, Cambridge, UK). Finally, the reactive bands were detected by chemiluminescence (SuperSignal, West Femto, Pierce, Washington, USA). The mouse polyclonal anti β-Tubulin antibody (1:1000 dilution; Sigma, Milan, Italy) was used to verify and ensure the correctness of the test.

Treatment: Resiniferatoxin (RTX, potent analog of capsaicin, which is an agonist at vanilloid receptors), JWH-133 (potent CB2 selective agonist), iodoresiniferatoxin (I-RTX, selective TRPV receptor antagonist) and AM630 (CB2 receptor antagonist) were purchased from Tocris Bioscience (Bristol, UK) [11]. These drugs were dissolved in dimethyl sulfoxide (DMSO). The final concentration of DMSO in cell cultures was 0.01%, while drug concentrations were RTX (5 μM), JWH-133 (100 nM), I-RTX (2.5 μM) and AM630 (10 μM). The samples were analyzed after 24 h treatment.

## 5. Conclusions

Paget’s disease of bone is a clinical condition with a not completely clear etiopathogenesis. Stronger efforts are needed to clarify the role of the EC/EV system in the pathophysiology of PDB and whether this interplay might open new therapeutic scenarios. To date, clinical studies about the effect of the modulation of EC/EV in patients with PDB are not yet available. In our study, we demonstrated that the modulation of OCs activity, through agents targeting cannabinoid receptors, could have beneficial effects on bone resorption. This strategy might represent an additional resource for implementing the management of PDB patients by modulating bone turnover and improving pain control.

## Data Availability

The data presented in this study are available on reasonable request from the corresponding author.
